# Machine Learning Customized Novel Material for Energy‐Efficient 4D Printing

**DOI:** 10.1002/advs.202206607

**Published:** 2023-02-05

**Authors:** Chaolin Tan, Qian Li, Xiling Yao, Lequn Chen, Jinlong Su, Fern Lan Ng, Yuchan Liu, Tao Yang, Youxiang Chew, Chain Tsuan Liu, Tarasankar DebRoy

**Affiliations:** ^1^ Singapore Institute of Manufacturing Technology Agency for Science, Technology and Research (A*STAR) 2 Fusionopolis Way Singapore 138634 Singapore; ^2^ Department of Materials Science & Engineering City University of Hong Kong Hong Kong SAR China; ^3^ Department of Materials Science & Engineering Pennsylvania State University University Park PA 16802 United States

**Keywords:** 4D printing, additive manufacturing, green metals, intrinsic heat treatment, machine Learning, new materials, sustainable materials

## Abstract

Existing commercial powders for laser additive manufacturing (LAM) are designed for traditional manufacturing methods requiring post heat treatments (PHT). LAM's unique cyclic thermal history induces intrinsic heat treatment (IHT) on materials during deposition, which offers an opportunity to develop LAM‐customized new materials. This work customized a novel Fe–Ni–Ti–Al maraging steel assisted by machine learning to leverage the IHT effect for in situ forming massive precipitates during LAM without PHT. Fast precipitation kinetics in steel, tailored intermittent deposition strategy, and the IHT effect facilitate the in situ Ni_3_Ti precipitation in the martensitic matrix via heterogeneous nucleation on high‐density dislocations. The as‐built steel achieves a tensile strength of 1538 MPa and a uniform elongation of 8.1%, which is superior to a wide range of as‐LAM‐processed high‐strength steel. In the current mainstream ex situ 4D printing, the time‐dependent evolutions (i.e., property or functionality changes) of a 3D printed structure occur after part formation. This work highlights in situ 4D printing via the synchronous integration of time‐dependent precipitation hardening with 3D geometry shaping, which shows high energy efficiency and sustainability. The findings provide insight into developing LAM‐customized materials by understanding and utilizing the IHT‐materials interaction.

## Graphical abstract

Existing additive‐manufactured commercial metallic powders require time‐ and energy‐consuming post heat treatments (PHT). The unique intrinsic heat treatment (IHT) in additive manufacturing offers an opportunity for material innovations. This work customized a “green metal” by machine learning, highlighting the high feasibility of 4D printing via synchronous integration of time‐dependent precipitation hardening with 3D geometry shaping.

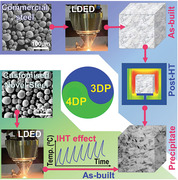

## 1 Introduction

The materials, structure, process, and performance are the critical aspects of enhancing the multifunctionality of additive manufacturing (AM) processed components.^[^ ^1^, ^2^ ^]^ Thus, the booming of new materials for AM is essential to advance the maturity and sustainability of AM technologies.^[^ ^3^ ^]^ However, existing commercial metallic powders for AM are designed and optimized for the conventional processing route (e.g., casting, hot isostatic pressing, spark plasma sintering, etc.) and might not be optimal for AM. Moreover, AM‐processed commercial metallic materials mostly require post heat treatments (PHT) to achieve good performance, which costs energy and emits CO_2_. The metallurgical industry is one of the largest emitters of greenhouse gases,^[^ ^4^ ^]^ of which heat treatment takes up a significant portion.^[^ ^5^ ^]^ Hence, sustainable alloy design to exempt PHT is a good strategy for making “green” metallic materials.

Laser additive manufacturing (LAM) typically includes laser‐directed energy deposition (LDED) and laser powder bed fusion (LPBF), which forms objects incrementally from point to line, layer, and finally to 3D component using the high‐energy laser to melt powder particles following a pre‐designed computer‐aided design (CAD) model and toolpath.^[^ ^6^ ^]^ The time‐temperature profile experienced by a part produced by LAM is very different from that produced by conventional manufacturing.^[^ ^7^ ^]^ During the LAM process, the material will experience unique thermal histories, including cyclic rapid heating and cooling. After the deposited material rapidly cools down from the liquid state, it will experience cyclic reheating when depositing adjacent tracks and subsequent layers.^[^ ^8^ ^]^ This cyclic reheating induces many short temperature spikes, leading to an intrinsic heat treatment (IHT) to the as‐deposited material.^[^ ^9^ ^]^

The IHT effect, when appropriately controlled, may trigger clustering or nucleation of hardening precipitates during the LAM process without additional PHT. For instance, the IHT effect on the deposited materials promoted the intrinsic formation of high‐density *β*′‐NiAl nanoparticles in LDED Fe‐19Ni‐*x*Al alloy^[^ ^8^ ^]^ and nanosized *η*‐Ni_3_Ti precipitates in LDED Fe‐19Ni‐5Ti alloy.^[^ ^9^ ^]^ Apart from steels, the precipitates triggered by the IHT effect were also reported in LDED of commercial magnesium and aluminum alloys (e.g., in situ formation of *β*′‐Mg_7_Gd precipitate in Mg–Gd magnesium alloy^[^ ^10^ ^]^ and Al_3_(Sc, Zr) precipitates in Al–Sc–Zr aluminum alloy^[^ ^11^ ^]^). Further straightforward investigation and understanding of solid‐state thermal cycling‐driven evolution of precipitates have been conducted recently, where the precipitation behavior of oxide and nonoxide precipitates in an LDED‐built 316L austenitic steel during cooling/heating cycles (with cooling and heating rates up to 4000 k s^−1^) were in situ observed by using transmission electron microscopy.^[^ ^12^ ^]^ However, this in situ precipitate behavior was observed in commercial materials, and there lacks work to customize new materials by fully exploiting the IHT to exempt PHT. To this end, the development of LAM‐customized new materials with in situ precipitation strengthening capability to potentially eliminate PHT by utilizing unique thermal history in LAM deposition provides insights into the booming of new materials for LAM.

Maraging steel is a martensitic age‐hardening advanced steel which is strengthened by the precipitation of intermetallics (e.g., *η*‐Ni_3_Ti, Ni_3_Mo, Ni_3_Al, NiAl, etc.) after heat treatment at about 450–550 °C for 3–9 h.^[^ ^8^, ^13^ ^]^ As a typical precipitation hardening ultrahigh strength steel, maraging steels are generally used as landing gear, helicopter undercarriages, rocket motor cases, and other applications which require a high strength‐to‐weight ratio.^[^ ^14^ ^]^ The Ni_3_Ti is the predominant precipitate in Ti‐containing maraging steels due to its high precipitation kinetics.^[^ ^13^, ^15^ ^]^ Hence, the Ni_3_Ti could be the promising precipitate in materials triggered by the IHT effect. To design lightweight maraging steel with Ni_3_Ti precipitation, the Fe–Ni–Ti–Al system was selected. Machining learning (ML) is an artificial intelligence technique that enables a machine or system to learn from data and make reliable decisions or predictions,^[^ ^16^, ^17^ ^]^ which has demonstrated promising capability in accelerating the design and discovery of new advanced materials.^[^ ^18^, ^19^ ^]^ Hence, to fully utilize the thermal history‐material interaction during LAM, this work implements ML to discover a Fe–Ni–Ti–Al novel maraging steel (NMS) with fast precipitation kinetics, which gains in situ precipitation hardening capability during LAM and eliminates the time‐consuming PHT. This ML customized NMS is an environmental‐friendly and sustainable material since it could reduce energy consumption and CO_2_ emission associated with materials PHT.

## 2 Machine Learning Assisted Material Customization

**Figure 1:**
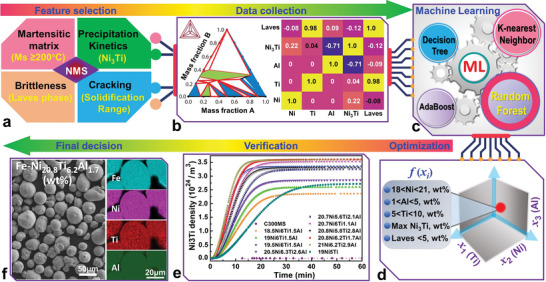
The schematic of machine learning (ML) assisted composition design of Fe–Ni–Ti–Al novel maraging steel (NMS). a) Feature selections in the design of NMS, b) data collections from Thermo‐Calc software and the correlation matrix of the input composition (Ni, Ti, and Al) and output (Ni_3_Ti precipitate and Laves phase weight fractions) in the surrogate models, c) ML by various algorithms (Random Forest is the most accurate one), d) composition optimization for the allowable range of alloying elements, e) time‐dependent dynamic precipitation behaviours of different compositions at 490 °C (the balance is Fe), and f) final decisive composition as Fe‐20.8Ni‐6.2Ti‐1.7Al (wt%) along with the morphology and elemental mapping of the produced powder.

The computational workflow of designing the new Fe–Ni–Ti–Al precipitation hardening NMS is summarized in **Figure** **1**. The phase contents (e.g., Ni_3_Ti precipitate and Laves phase) and critical temperatures (e.g., martensite transformation temperature and solidification range) were selected as the key features for thermodynamics modelling and alloy composition optimization (Figure 1a). In the data collection stage, a Design for Computer Experiment (DoCE) table was first generated with many randomized compositions as inputs. After that, the CALculation of PHAse Diagrams (CALPHAD) models were created and solved in Thermo‐Calc software automatically in batches for all compositions within the DoCE table (Figure 1b). The CALPHAD results obtained in the DoCE were used to train surrogate models by ML, enabling rapid exploration of the alloy design space in the subsequent composition optimization stage. The surrogate models took any alloy composition as input and predicted the values of the key features (e.g., Laves phase and Ni_3_Ti precipitate).

**Figure 2:**
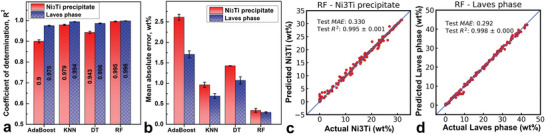
Performance comparison of different ML models for Laves phase and Ni_3_Ti precipitate surrogate modelling with a) coefficient of determination (i.e., *R* ^2^ score) and b) mean absolute error (MAE). Ground truth data plotted against predicted data points by RF regression model for c) Laves phase and d) Ni_3_Ti precipitate prediction, demonstrating great capability of predicting phase content given the alloy composition. (Note: the higher *R* ^2^ score indicates better performance, while a lower MAE value means better performance.).

In the surrogate modelling, four ML algorithms, i.e., Decision Tree (DT), K‐nearest Neighbour (KNN), Adaptive Boosting (AdaBoost), and Random Forest (RF), were trained and tested. Comparison of tested performance of various ML models for Laves phase and Ni_3_Ti precipitate prediction are shown in **Figure** **2**a,b. The most accurate one, i.e., RF, in this case, was selected (Figure 1c) as the surrogate model. The ground truth values plotted against predicted values by the RF regression model in Figure 2c,d yield *R* ^2^ scores greater than 99%, showing an excellent capability of predicting values close to the ground truth values of Laves phase and Ni_3_Ti precipitate contents given a particular alloy composition. The ML model performance evaluations for other surrogate models (i.e., KNN, DT, and AdaBoost) are presented in Figure S1 in the Supporting Information. More details on ML model training, hyperparameter tunings, and data descriptions are provided in the Text (Supporting Information) titled “Discussion on the machine learning algorithms for surrogate models”.

Once the surrogate models were established, they were used to formulate the composition optimization problem. The design space or the allowable range of the alloying elements (i.e., [wt% Ti_min_, wt% Ti_max_], [wt% Ni_min_, wt% Ni_max_], [wt% Al_min_, wt% Al_max_]), the constraint functions (i.e., wt% Al < wt% Ti; Laves < Laves_max_ wt%), and the objective function (i.e., maximizing Ni_3_Ti and minimizing Laves content) were specified in the optimization problem (Figure 1d). The Ni content in commercial C300 maraging steel (CMS) is about 18 wt%.^[^ ^13^ ^]^ To increase the fraction of Ni_3_Ti, the amount of Ni in the customized steel should be >18 wt%. The high Ni content ensures the decomposition of austenite and the formation of only martensite, even at low cooling rates. However, there is also an upper limit for Ni content since the high Ni will increase the instability of martensite at elevated temperatures and promote the formation of retained austenite. Besides, as shown in Figure S2a (Supporting Information), the martensite transformation temperature decreases with increasing Ni content. To secure the martensitic matrix, the martensite transformation finish temperature (i.e., when 90% martensite is formed) is deemed reliable at a value higher than 200 °C, considering the heat accumulation during multiple layers laser depositions. Furthermore, the Schaeffler–Delong diagram in Figure S2b (Supporting Information) also indicates that the high Ni content will increase the austenite phase, as referred to as commercial C300 maraging steel. Hence the upper limit for the Ni is set as 21 wt%, i.e., 18 < Ni < 21 wt%. The correlation matrix in Figure 1b indicates that the increase of Ti will increase the Fe_2_Ti Laves phase fraction, and the high Al content will suppress the formation of Ni_3_Ti precipitate. Hence, the upper limit of Ti was set as 10 wt%, considering that the stoichiometric ratio of 3:1 for Ni:Ti could benefit Ni_3_Ti formation. Thus, the Ti content is set as 5 < Ti < 10 wt%. For the volatile Al, the minimum content is set as 1 wt%, and the up limit is designed as less than Ti, i.e., 1 < Al < 5 wt%.

**Figure 3:**
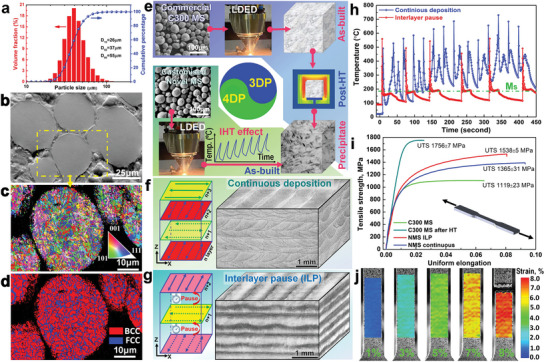
Overview of the machining learning customized powder, LDED processes and mechanical properties of the customized Fe–Ni–Ti–Al NMS. a) Powder particle size distributions. b) Cross‐sectional view, c) inverse pole figure, and d) phase distribution map of powder. e) Illustrative comparison between 3D printing (**3DP**) of CMS followed by PHT to form precipitates, and 4D printing (**4DP**) of ML customized NMS with in situ formed massive precipitates incited by IHT without PHT. Spatial morphologies of LDED‐processed NMS by f) continuous deposition and g) interlayer pause (ILP) deposition strategies. h) Time‐dependent thermal histories in continuous and ILP deposition strategies (the *M*_s_ = 182 °C). i) Tensile engineering stress–strain curves of NMS (as‐built) and CMS (repeated tensile tests are plotted in Figure S4a in the Supporting Information). j) Deformation behaviour of NMS by in situ DIC monitoring.

A population‐based metaheuristic algorithm, i.e., Differential Evolution,^[^ ^20^ ^]^ was used to solve the optimization problem. By adjusting the settings in the above design space and constraint functions, one can obtain a shortlisted set of compositions. And then, by verifying and comparing these shortlisted compositions in material kinetics simulation using precipitation module “TC‐PRISMA” in Thermo‐Calc software, one can estimate the time‐dependent dynamic precipitation behaviors (e.g., Ni_3_Ti precipitation rate and maximum content as shown in Figure 1e), thus reaching the final composition decision Fe‐20.8Ni‐6.2Ti‐1.7Al (wt%). The morphology along with elements mapping of the produced powder are included in Figure 1f, which is in a good spherical shape with an average particle size of about 37 µm (**Figure** **3**a). This composition enables rapid precipitation of Ni_3_Ti with an extremely high density of 3.3×10^24^ m^−1^ (approaching peak density) after only 15 min of heat treatment at 490 °C. The brittleness and printability (e.g., cracking tendency) of this customized NMS are also evaluated and compared with the CMS. The solidification temperature range (critical indicator for solidification cracking) of NMS shares a similar value with that in CMS, as shown in Figure S2c (Supporting Information), suggesting a low cracking possibility during LDED since the CMS demonstrated excellent weldability.^[^ ^13^ ^]^ Furthermore, the predicted Laves phase is below 2 wt% in this NMS, which is slightly lower than the C300 CMS (see Figure S2d in the Supporting Information). Details on the composition optimization problem and solution are presented in the Text (Supporting Information) titled “Discussion on the alloy composition optimization”. The Python program for ML surrogate modelling and alloy composition optimization is available in the Source Code in the Supporting Information.

## 3 Results and Discussion

### 3.1 Conception and Overview of 4D Printing of Customized Steel

To evaluate the densification quality of the powder, the cross‐sectional view of the powders is displayed in Figure 3b, which reveals high‐density powder particles without evident internal defects. The EBSD analysis of the powder particles is displayed in Figure 3c,d. The inverse pole figure of the powder in Figure 3c suggests a random grain orientation without evident texture. The phase distribution map in Figure 3d indicates that the powder has major body‐centered cubic (BCC) martensite with minor face‐centered cubic (FCC) austenite segregation in the cellular structure boundaries.

As illustrated in Figure 3e, the LDED‐processed CMS parts need to undergo age treatment for 3–9 h to form the precipitates for strengthening.^[^ ^13^ ^]^ In contrast, the ML customized NMS can in situ form massive precipitates without undergoing PHT, suggesting a synchronous integration of 3D geometry shaping with heat treatment. This integration can be considered 4D printing since the time‐consuming aging heat treatment (time dimension) was incorporated during 3D printing (i.e., 3D geometry + time dimension). The classic concept terms 4D printing as: the shape, property, or functionality of a 3D printed structure can change as a function of time when subjected to different environmental stimuli (e.g., heat, light, water, etc.).^[^ ^21^ ^]^ Current mainstream 4D printing can be termed ex situ 4D printing since the time‐dependent evolutions occur after 3D objective formation rather than synchronously.^[^ ^22^ ^]^ In contrast, this work highlights in situ 4D printing via in situ integration of time‐dependent evolution (i.e., precipitation) with 3D geometry shaping. Two key factors to accomplish this in situ 4D printing: (i) The unique thermal history leads to an IHT effect on the as‐deposited material.^[^ ^9^ ^]^ (ii) The ML‐customized NMS has a fast kinetic of precipitation with a high precipitation rate (Figure 1e), which enable the formation of precipitates within a short time.

As thermal history is tunable by varying the processing strategies, two deposition strategies (i.e., continuous and interlayer pause (ILP), as described in experimental section) were designed. The schematics of two deposition strategies and the spatial microstructures of the deposited samples are displayed in Figure 3f,g. The continuous sample shows uniform macrostructure, while the ILP sample displays layerwise features consisting of alternated dark and white layers. The distinct microstructures are attributed to the different thermal histories in two deposition strategies, as shown in Figure 3h. The continuous deposition led to thermal accumulation in the deposited materials, which makes the temperature in solidified material higher than the martensite start temperature *M*_S_ (i.e., 182 °C as shown in Figure S3 in the Supporting Information). In contrast, the ILP enables the solidified material to cool down below *M*s, which means more BCC martensitic phases will be formed in the ILP sample.

The representative engineering stress–strain curves of the 4D printed NMS and 3D printed CMS are displayed in Figure 3i, with the correlative results being summarized in Table S2 in the Supporting Information. The NMS deposited by ILP shows higher yield strength (YS) and ultimate tensile strength (UTS) than the continuous sample while sharing similar elongation (El). Besides, the as‐built NMS, especially the ILP sample, shows much higher UTS (more than 400 MPa) than the as‐built CMS. Notably, the uniform El (more important than break elongation for engineering applications) of the as‐built NMS is also much higher than that of the as‐built and post aged CMS. The achieved mechanical properties of NMS are also compared with the AM‐processed CMS in the literature (see Figure S4b in the Supporting Information). The as‐built CMS has a UTS below 1.25 GPa in general. Although aging PHT can enhance the UTS of CMS up to 2.2 GPa, their uniform El and break El are generally below 5% and 5.6%, respectively.^[^ ^23^, ^24^, ^25^, ^26^, ^27^, ^28^ ^]^ In contrast, the 4D printed NMS achieved a UTS of about 1.54 GPa alongside an El of 8.1%, highlighting the superior strength‐ductility combination to CMS. Figure 3j exhibits the deformation behavior of the NMS sample observed by digital image correlation (DIC) at different global strain stages. The inhomogeneous deformation with multiple strain localization bands is observed when the strain reaches 3% and higher. This is distinct from uniform deformation materials, whereby higher strain localizes only near the necking region prior to material fracture.

### 3.2 Microstructures and In Situ Precipitation Mechanisms

**Figure 4:**
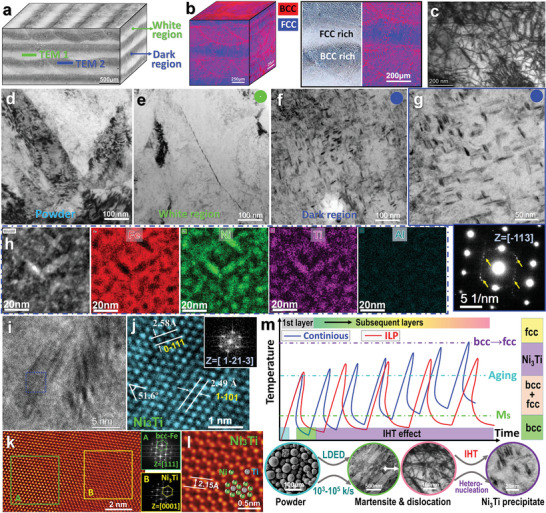
Microstructure analysis of the powder and LDED‐processed NMS by ILP deposition strategy. a) Spatial distribution of dark and white layers (FIB extracted regions for TEM testing are illustrated). b) Spatial distribution of BCC and FCC phases. c) TEM image shows high‐density dislocations in the ILP sample. d) Powder and e) white regions of the ILP sample show no evidence of precipitates. f) TEM observations on the dark regions show massive precipitates. g) Zoom‐in image of acicular precipitates and correlative SADP. h) EDS mapping analysis on the precipitates. i,j) HR‐TEM analysis on the Ni_3_Ti precipitates, where (j) is a closer view of the labelled zone in (i). k) High‐resolution HAADF STEM images and FFTs of FCC‐Fe matrix and round precipitate. l) High‐resolution HAADF‐STEM image showing periodic Ti and Ni atoms of Ni_3_Ti. m) Schematic shows in situ precipitation behaviour and mechanism.

Overall, the LDED‐processed NMS displayed multiscale hierarchical microstructures. In the macro‐scale, there is an alternating distribution of dark and white layers in the entire sample (**Figure** **4**a). Within the layers, there are periodic melt pools. Upon examination within the melt pools, there are cellular and dendritic structures with size/arm spacing below 10 µm, which were observed in both dark and white regions of the LDED‐processed NMS sample. The spatial phase distributions are illustrated in Figure 4b, in which the BCC‐rich regions match with the dark layers. The alternating BCC‐rich and FCC‐rich in different deposition layers form macro‐scale layerwise dual‐phase. Interestingly, a closer view of the BCC‐rich layer revealed microscale FCC and BCC dual‐phase microstructures. The layerwise heterostructured phases could be attributed to the different cooling rates during material solidification (as illustrated in Figure S5 in the Supporting Information), where the melt pool boundaries and layer interfaces experienced a higher cooling rate than other regions. The high cooling rates benefit the formation of the BCC phase. The dislocation features in bulk ILP samples were examined by TEM (Figure 4c), which reveals high‐density dislocation tangles due to the intensified residual stress caused by the high cooling rate associated with the LDED process, especially with the ILP deposition strategy.

To investigate the in situ precipitation behavior, the TEM foils were extracted from the dark and white regions in the ILP sample (as illustrated in Figure 4a and Figure S6 in the Supporting Information) and from a powder particle by FIB for comparison. TEM morphologies of the powder (Figure 4d) and white region (Figure 4e) of the ILP sample suggest the absence of precipitate. In contrast, massive acicular precipitates with a length of about 5–50 nm are observed in the dark region of the ILP sample, as shown in Figure 4f,g. The inset SADP in Figure 4g shows super‐lattice diffraction spots, where the large spots come from BCC‐Fe while the weak spots are from the precipitates. The low‐magnification observations on the dark region in Figure S7 (Supporting Information) indicate these precipitates are prevalent in both the dislocation accumulation and dislocation‐lean regions in the BCC‐Fe matrix (Ni‐rich martensite), i.e., in situ formation of precipitates in the entire dark region in ILP sample. The EDX mapping in Figure 4h indicates that the precipitates are rich in Ti and Ni elements, which is likely to be Ni_3_Ti since the close‐packed hexagonal (hcp) *η*‐Ni_3_Ti is the main precipitate phase responsible for the strengthening of the maraging steels containing Ti.^[^ ^14^, ^15^ ^]^ Further analysis of acicular precipitates in Figure 4i by fast Fourier transform (FFT) and HRTEM analysis in Figure 4j confirm the Ni_3_Ti precipitates. Close STEM observation on the precipitate–matrix interface is displayed in Figure 4k, where the BCC‐Fe matrix and hcp‐structured precipitate are identified by the FFTs, and a transitional interface with good coherency between them was observed. The HAADF‐STEM in Figure 4l (taken from zone B in Figure 4k) shows the Ti and Ni atoms distributions observed from the [0001] zone axis, confirming the hcp‐Ni_3_Ti precipitate. Note that the dark atoms in Figure 4l are supposed to be Ti since contrast in HAADF‐STEM imaging originates from low atomic numbers (*Z*).

The in situ formation process and mechanism of Ni_3_Ti precipitates are illustrated in Figure 4m. There are several contributing aspects to accomplishing this in situ precipitation. (i) Materials and precipitation kinetics: The ML‐optimized materials composition increased precipitation rate and density (Figure 1), which facilitates instant precipitation without PHT. Furthermore, the interaction between Ni and Ti to form hexagonal *η*‐Ni_3_Ti is the most rapid reaction during aging, and Ni_3_Ti is the main precipitate phase responsible for strengthening the maraging steels containing Ti.^[^ ^14^, ^29^, ^30^ ^]^ Some maraging steels can be hardened even only after 5 s at 550 °C HT, showing an increase in hardness by 160% (i.e., from 310 to 490 VHN) due to the coclustering between Ti–Ni, Ti–Al, and Ti–Mn.^[^ ^14^ ^]^ (ii) Process and thermal history control: During LDED, the IHT effect and thermal accumulation will affect the microstructures significantly. The thermal accumulation in the continuously deposited sample inhibits the solidified material from rapid cooling to a temperature below the *M*_s_ (i.e., 182 °C), suppressing martensite formation. In contrast, the ILP deposition strategy enabled the solidified materials to cool down to *M*_s_, facilitating martensite formation. Note that the martensitic matrix is essential for the precipitate formation as it contains high‐density dislocations.^[^ ^13^ ^]^ The subsequent layers deposition induced cyclic IHT to the solidified materials, which contains multiple temperature spikes up to 550 °C (Figure 3h), reaching the aging temperature (about 500 °C as parsed by DSC in Figure S8 in the Supporting Information). Notably, the temperature spikes coupled with thermal accumulation in the continuous deposition are likely to promote the austenite reversion since the attached temperature can reach the complete BCC to FCC transformation temperature (around 640 °C as in Figure S8 in the Supporting Information), which also reduces the FCC fraction in the material. (iii) Solidification and dislocation: The LDED has a very high cooling rate of 10^3^–10^5^ k s^−1^,^[^ ^1^ ^]^ and the ILP deposition further enhances the cooling rate of materials, which induces high‐density dislocations (as disclosed in Figure 4c) in the solidified materials. The dislocations will interact with solute and precipitate nucleation. (iv) Heterogeneous nucleation: Prior to the nucleation of these precipitates, there is usually a tendency for solute segregation at the dislocations. The crystal defects (e.g., dislocations and grain boundaries) change the solute diffusion kinetics since the dislocations and grain boundaries are fast diffusion paths.^[^ ^31^ ^]^ Hence, nucleation at structural defects occurs faster than homogeneous nucleation, and heterogeneous precipitation facilitates the formation of more stable phases. The dislocations favor nucleation by causing a bias in the energy balance and reducing the energy barrier.^[^ ^32^ ^]^ The dense dislocations in the ILP sample provided more nucleation sites for Ni_3_Ti precipitates. Besides, the ILP sample experienced a higher cooling rate (especially for the interfacial regions) than the continuous deposition, which could enhance the degree of undercooling of the melt pool, and also enhance the nucleation rate of the precipitates.^[^ ^33^ ^]^ Consequently, the interplay between materials composition, LDED deposition strategy, high‐density dislocation martensitic matrix and IHT effect facilitated the in situ formation of Ni_3_Ti nanoprecipitates via heterogeneous nucleation.

### 3.3 Correlation Between Precipitation and Mechanical Property

**Figure 5:**
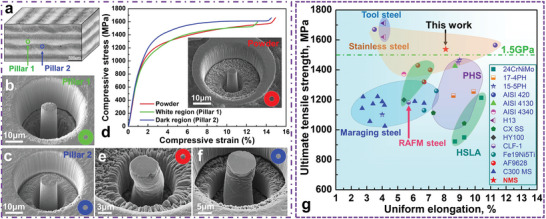
a) The illustration shows the locations of micro‐pillars in the ILP sample. b,c) Morphologies of micropillars extracted from the white (pillar 1) and dark (pillar 2) regions, respectively. d) Compressive stress–strain curves of pillars taken from the ILP sample (white and dark regions) and raw powder. e,f) Fracture morphologies of the powder and dark region micropillars, respectively. g) Tensile properties of 4D printing NMS in this work compared with those of a wide range of AM‐processed high‐strength steels (all in as‐built condition), including: (i) high‐strength low‐alloy steels (HSLA), such as 24CrNiMo,^[^ ^34^ ^]^ AF9628,^[^ ^35^ ^]^ and HY100;^[^ ^36^ ^]^ (ii) precipitation hardening steels (PHS), such as 17‐4PH,^[^ ^37^, ^38^ ^]^ 15‐5PH,^[^ ^39^, ^40^ ^]^ and CX SS;^[^ ^41^, ^42^ ^]^ (iii) high‐strength stainless steels, such as AISI 420,^[^ ^43^, ^44^ ^]^ AISI 4130,^[^ ^45^ ^]^ and AISI 4340;^[^ ^46^ ^]^ (iv) tool steel (e.g., H13^[^ ^47^ ^]^), (v) reduced‐activation ferritic/martensitic (RAFM) steel (e.g., CLF‐1^[^ ^48^ ^]^); and (vi) maraging steels, such as Fe19Ni5Ti^[^ ^9^ ^]^ and C300MS.^[^ ^23^, ^24^, ^25^, ^26^, ^27^, ^28^ ^]^

The micropillar compression on localized regions of the NMS bulk material and powder was conducted to further investigate the effect of in situ formed precipitates on mechanical properties. As illustrated in **Figure** **5**a, the micropillars (Figure 5b,c) were extracted from the dark and white regions in the bulk ILP sample (positioned by Vickers indentations as shown in Figure S6g in the Supporting Information), together with the micropillar of the NMS powder particle (Figure 5d) by FIB. The measured compressive stress‐strain curves are displayed in Figure 5d, demonstrating that the dark region achieved a higher strength than both the white region and powder. Notably, the white region shows a similar strength to the powder due to the lack of precipitates (see Figure 4). The higher strength associated with the dark region could originate from the high‐density dislocations and precipitates as they could strengthen the material. Hence, understanding localized mechanical properties are essential to clarify the in situ precipitation behavior and its effect on mechanical properties. The pillars from the powder (Figure 5e) and dark region (Figure 5f) both exhibit ductile fracture behavior without evident shearing cracks, which suggests the nanoprecipitates strengthened materials without an evident increase in brittleness.

### 3.4 Mechanism of High Strength‐Ductility Synergy

The achieved mechanical properties of NMS in this work are compared with a wide range of AM‐processed high‐strength steels (HSSs) in as‐built conditions without PHT. It is challenging for HSSs to achieve UTS higher than 1.5 GPa without PHT. The customized NMS exhibited UTS higher than 1.5 GPa and a uniform El higher than 8%, which is one of the excellent balances of strength‐uniform El among different types of steels, as summarized in Figure 5g. The underlying mechanisms accounting for high strength are analyzed in the Experimental Section. The fine lath martensites (see Figure S7a in the Supporting Information) led to grain boundary strengthening (*σ*_g_) following the Hall–Petch mechanism,^[^ ^49^ ^]^ contributing about 285 MPa to the yield strength. Additionally, as disclosed by TEM results (Figure 4c), the high dislocation density (*ρ*) in the NMS sample (calculated as *ρ* = 1.77 × 10^14^ m^−2^) also contributed about 307 MPa to the YS via dislocation strengthening (*σ*_d_) following the Taylor hardening law.^[^ ^50^ ^]^ Furthermore, the 5–50 nm sized Ni_3_Ti precipitates (Figure 4g) are strong enough to resist the dislocations' movement, strengthening the matrix following the Orowan bowing mechanism.^[^ ^15^ ^]^ The strengthening by Ni_3_Ti is calculated as about 1118 MPa, and the dark region takes up about 38% of the whole sample (Figure 4). Hence, the contribution of Ni_3_Ti strengthening in the entire sample is about 425 MPa. Consequently, the total strengthening is calculated as 1017 MPa, which is close to the measured YS of 966 MPa.

The multiple deformation bands (also considered as Luders band) in NMS (see Figure 3j) during tensile are feasible in delaying the necking and improving the ductility.^[^ ^51^ ^]^ Besides, the dual‐phase structures could also experience the transformation‐induced plasticity (TRIP) effects during tensile tests, where the FCC austenite could transform to BCC martensite transformation upon deformation and contribute to El improvement.^[^ ^52^ ^]^ This was demonstrated by Figure S9 (Supporting Information), where the BCC phase fraction increased after the fracture compared with the undeformed condition. Furthermore, the synergetic deformation and dynamic strain partition between the soft austenite and hard martensite regions could suppress the strain localisation at the phase interface, which retards the crack initiation and contributes to the large uniform El.^[^ ^53^ ^]^

## 4 Conclusions

In summary, this work customized a novel maraging steel by machine learning, which enabled the in situ formation of precipitates during LDED without needing PHT. The ILP deposition strategies promoted the formation of a martensitic matrix with high‐density dislocations and created a hierarchical dual‐phase. The fast precipitation kinetics of the material and the unique IHT effect in LDED promoted in situ precipitation of massive nano‐Ni_3_Ti. The localized mechanical properties evaluated by micropillar compressions demonstrated that the in situ formed Ni_3_Ti precipitates enhanced the strength of LDED‐processed NMS compared to the feedstock powder. The as‐built NMS achieved tensile strength of about 1.54 GPa along with a uniform elongation of 8.1%, which is superior to a wide range of as‐AM‐processed high‐strength steels. This work highlighted the potential approach to developing high‐performance metals by leveraging the unique thermal history of laser AM, thus further boosting AM‐customized new materials with improved functionality and sustainability.

## 5 Experimental Section

### Feedstock Powder Material

The machine learning (ML) designed Fe‐20.8Ni‐6.2Ti‐1.7Al (wt%) novel maraging steel (NMS) powder was processed by gas atomization. As summarized in Table S1 (Supporting Information), the measured compositions of the gas‐atomized powders by inductively coupled plasma atomic emission spectroscopy (ICP‐AES) are Fe‐21.1Ni‐6.3Ti‐1.3Al (wt%), which is close to the designed value with minor deviation. The oxygen (O) and carbon (C) of the powder, as measured by ICP‐AES, are 0.024 and 0.012 wt%, respectively. The powder particle size distributions were measured by a laser scattering particle size analyzer. The *D*_10_, *D*_50_, and *D*_90_ of the powder are 26, 37, and 55 µm, respectively. The cross‐sectional view of the powders was observed by field emission scanning electron microscope (FE‐SEM) and electron back‐scatter diffraction (EBSD) analysis to examine powder densification, grain orientation, and phase distributions. The martensite start temperature (*M*_s_) of this NMS is measured by using a Dilatometer experiment using the heating and cooling rates of 10 k min^−1^. The commercial C300 maraging steel (CMS) with a composition of Fe‐18.3Ni‐9.1Co‐4.9Mo‐0.75Ti‐0.1Si‐0.09Cr‐0.04Mn‐0.01C, wt%) and with an average particle size of about 40 µm, was also processed by LDED for benchmarking.

### Additive Manufacturing

The customized powder was processed by using the powder‐blown LDED system developed by the Singapore Institute of Manufacturing Technology (SIMTech). The optimized LDED process parameters for NMS are laser power 905 W, scan speed 1000 mm min^−1^, hatch space 0.65 mm and powder feeding rate 2.3 g min^−1^. A raster laser scan pattern with 90° interlayer rotation was used, and the resultant layer thickness is about 0.4 mm. Almost fully dense NMS blocks with a relative density ≥99.9% were obtained. The bulk NMS samples for microstructures observation and mechanical properties testing were deposited in two strategies: (i) continuous deposition strategy, i.e., without pause between layers, and (ii) interlayer pause (ILP) deposition strategy, i.e., implement a pause every layer to enable the deposited materials to cool down to 50–60 °C. The thermal histories in these two LDED deposition strategies were recorded by a thermocouple attached to the edge of the first deposited layer. The optimized LDED process parameters for CMS, i.e., laser power, scan speed and hatch space, for CMS are 850 W, 1200 mm min^−1^ and 0.8 mm, respectively. The LDED‐built CMS was aged at 490 °C for 4 h in a resistance furnace for precipitation hardening, followed by cooling in the air.

### Microstructural Characterization

The as‐built samples were cut along the *Z* (build direction), *Y*, and *X* directions to study the macro‐morphologies using an OLYMPUS MX51 optical microscope (OM). The microstructures were observed by the FEI Helios NanoLab 600i SEM system (with energy dispersive spectrometer (EDS)) and EBSD along the build direction. The EBSD tests were conducted by an Oxford EBSD detector, using a step size of 50 nm at 20 kV. The inverse pole figure, band contrast maps, and phase distribution maps were analysed by commercial HKL Channel 5 software. Transmission electron microscope (TEM) samples were extracted from the specified regions of the as‐built samples using an FEI Scios dual‐beam Focused Ion Beam (FIB) system. An FEI Talos F200X TEM system operating at 200 kV was used to examine the multiscale precipitates using selected area diffraction pattern (SADP), scanning transmission electron microscopy (STEM), and energy‐dispersive X‐ray (EDX) spectroscopy analyses. The high‐resolution high‐angle annular dark field (HAADF) STEM observations were conducted on an FEI Titan Themis G2 instrument equipped with double spherical aberration correctors for probe forming and image forming lenses operated at 300 kV. The phase transformation temperature of NMS powder was identified by using a differential scanning calorimetry (DSC) system.

### Mechanical Testing

The microhardness was tested by a MATSUZAWA MMT‐X3 microhardness tester using a load of 100 gf and a dwelling time of 15 s. The average value was determined from more than 20 measurements. The tensile samples were extracted from the built block horizontally. The dimensions of the reduced sections in the tensile coupon are 6 mm in width, 3 mm in thickness, and 26 mm in length. Tensile tests were conducted on an INSTRON 5982 universal testing machine with a 1 mm min^−1^ loading speed. A noncontact advanced video extensometer (AVE) was applied to measure the tensile strain, and a primary gauge length of about 20 mm was used. A 2D digital image correlation (DIC) system was used for in situ monitoring of the deformation and failure behavior of samples during tensile tests. High‐resolution (2448 × 2048 pixel) images were taken at 5 frames s^−1^ (aperture size f/2.8 and exposure time 1/20 s) using a FLIR Grasshopper3 CCD camera. A Zeiss GOM Correlate Pro software was used to analyze those images to obtain the strain distribution maps.

Micropillar compression tests on the selected regions of the as‐built sample were carried out to understand the localized mechanical properties. Micropillars were milled from selected regions of the as‐built sample and the powder by the focused ion beam (FIB) system using a Ga ion beam. The diameters of the micropillars for the powder and as‐built are about 5 and 9 µm, respectively, with a height‐to‐diameter ratio of about 3. The taper angles are all below 3°. The micropillar compressions were conducted with a Micro‐Materials NanoTest system using a *ϕ*20 µm flat diamond indenter. The loading and unloading rates are 1 and 10 mN/s, respectively. The deformed pillars were observed by SEM at a tilted angle of 40°.

### Strengthening Mechanisms Analysis

To understand the strengthening behavior in LDED processed ILP deposited NMS, here analyze the strengthening mechanism below. The fine lath martensites led to grain boundary strengthening (*σ*_g_) following the Hall–Petch mechanism

(1) $$\sigma_{g}=kD^{-1/2}$$

The *D* is the mean thickness of the lath martensites, which is about 273±67 nm, as quantified from the TEM images. The *k* is the strengthening coefficient and is estimated as 149 MPa·µm^1/2.[^ ^49^ ^]^

The higher dislocation density (*ρ*) in the NMS sample could also contribute to a high YS via dislocation strengthening (*σ*_d_) following equation^[^ ^50^ ^]^

(2) $$\sigma_{d}=M\beta Gb\sqrt{\rho }$$

where the Taylor factor *M* is about 2.9 for BCC Fe,^[^ ^54^ ^]^ and *β* is the constant coefficient of 0.5.^[^ ^50^ ^]^ The Burgers vector ***b*** and shear modulus G of the matrix in martensitic steel are 0.249 nm^[^ ^15^ ^]^ and 64 GPa,^[^ ^55^ ^]^ respectively. The dislocation densities were calculated as *ρ* = 1.77 × 10^14^ m^−2^ by using the line‐intercept method as elaborated by Norfleet et al.^[^ ^56^ ^]^

Additionally, as revealed in Figure 4, the Ni_3_Ti precipitates are strong enough to resist the dislocations' movement, leading to the strengthening of the matrix following the Orowan bowing mechanism^[^ ^15^ ^]^

(3) $$\left\{\begin{array}{c} \sigma_{o}=\frac{Gb}{2\pi K\left(\lambda -d\right)})\;\mathrm{In}\;\left(\frac{\lambda -d}{2b}\right)\\ \frac{1}{K}=\frac{1}{2}\;\left(\frac{1}{1-v}+1\right) \end{array}\right.$$

where *d* is the average diameter of the particles, *v* is Poisson's ratio of the matrix (i.e., *v* = 3 and *K* = 0.82), and *G* and ***b*** are the same as defined above. The Ni_3_Ti nanoprecipitates are about 4±0.8 nm in diameter and 17±5 nm in length on average, and it can be simplified as a sphere of an equivalent volume of diameter *d* = 7.5 nm. The *λ* is measured as about 15 nm on average.

## Conflict of Interest

The authors declare no conflict of interest.

## Author Contributions

C.T. proposed the study, led the project, carried out the main experiments, and wrote the paper. X.Y. conducted the machine learning together with L.C. and co‐wrote the paper. Q.L., T.Y. and C.T.L. conducted the FIB and TEM characterization. J.S. performed OM and SEM microstructure characterization. F.L.N. conducted the EBSD analysis. Y.L. performed micropillar compression tests. Y.C. performed the processing parameters optimization and provided resources for this work. T.D. contributed to machine learning and reviewed and edited the paper. All co‐authors contributed to the result discussions and commented on the manuscript.

## Supporting information

Supporting Information

Supporting Information

## Data Availability

The data that support the findings of this study are available from the corresponding author upon reasonable request.

## References

[1] D. Gu , X. Shi , R. Poprawe , D. L. Bourell , R. Setchi , J. Zhu , Science 2021, 372, eabg1487. 34045326 10.1126/science.abg1487

[2] Y. Yang , X. Song , X. Li , Z. Chen , C. Zhou , Q. Zhou , Y. Chen , Adv. Mater. 2018, 30, 1706539. 10.1002/adma.201706539 29920790

[3] C. Han , Q. Fang , Y. Shi , S. B. Tor , C. K. Chua , K. Zhou , Adv. Mater. 2020, 32, 1903855. 10.1002/adma.201903855 32431005

[4] D. Raabe , C. C. Tasan , E. A. Olivetti , Nature 2019, 575, 64. 31695209 10.1038/s41586-019-1702-5

[5] X. Zhang , K. Jiao , J. Zhang , Z. Guo , J. Cleaner Prod. 2021, 306, 127259.

[6] Y. Chew , Z. G. Zhu , F. Weng , S. B. Gao , F. L. Ng , B. Y. Lee , G. J. Bi , J. Mater. Sci. Technol. 2021, 77, 38.

[7] T. DebRoy , H. L. Wei , J. S. Zuback , T. Mukherjee , J. W. Elmer , J. O. Milewski , A. M. Beese , A. Wilson‐Heid , A. De , W. Zhang , Prog. Mater. Sci. 2018, 92, 112.

[8] P. Kürnsteiner , M. B. Wilms , A. Weisheit , P. Barriobero‐Vila , E. A. Jägle , D. Raabe , Acta Mater. 2017, 129, 52.

[9] P. Kürnsteiner , M. B. Wilms , A. Weisheit , B. Gault , E. A. Jägle , D. Raabe , Nature 2020, 582, 515. 32581379 10.1038/s41586-020-2409-3

[10] D. Zheng , Z. Li , Y. Jiang , R. Li , Y. Wu , Y. Tu , X. Cheng , P. Fu , L. Peng , H. Tang , Addit. Manuf. 2022, 57, 102957.

[11] P. Kürnsteiner , P. Bajaj , A. Gupta , M. B. Wilms , A. Weisheit , X. Li , C. Leinenbach , B. Gault , E. A. Jägle , D. Raabe , Addit. Manuf. 2020, 32, 100910.

[12] M. Ben Haj Slama , L. Yedra , E. Heripre , M. V. Upadhyay , Materialia 2022, 21, 101368.

[13] D. Hudok , Crit. Met. Handb. 1990, 1, 200.

[14] E. V. Pereloma , A. Shekhter , M. K. Miller , S. P. Ringer , Acta Mater. 2004, 52, 5589.

[15] C. Tan , K. Zhou , W. Ma , P. Zhang , M. Liu , T. Kuang , Mater. Des. 2017, 134, 23.

[16] T. DebRoy , T. Mukherjee , H. L. Wei , J. W. Elmer , J. O. Milewski , Nat. Rev. Mater. 2020, 6, 48.

[17] C. Wang , X. P. Tan , S. B. Tor , C. S. Lim , Addit. Manuf. 2020, 36, 101538.

[18] C. Zou , J. Li , W. Y. Wang , Y. Zhang , D. Lin , R. Yuan , X. Wang , B. Tang , J. Wang , X. Gao , H. Kou , X. Hui , X. Zeng , M. Qian , H. Song , Z.‐K. Liu , D. Xu , Acta Mater. 2021, 202, 211.

[19] P. Liu , H. Huang , S. Antonov , C. Wen , D. Xue , H. Chen , L. Li , Q. Feng , T. Omori , Y. Su , npj Comput. Mater. 2020, 6, 62.

[20] R. Storn , K. Price , J. Glob. Optim. 1997, 11, 341.

[21] F. Momeni , N. Mehdi Hassani , X. Liu , J. Ni , Mater. Des. 2017, 122, 42.

[22] X. Kuang , D. J. Roach , J. Wu , C. M. Hamel , Z. Ding , T. Wang , M. L. Dunn , H. J. Qi , Adv. Funct. Mater. 2019, 29, 1805290.

[23] C. Tan , K. Zhou , M. Kuang , W. Ma , T. Kuang , Sci. Technol. Adv. Mater. 2018, 19, 746. 10.1080/14686996.2018.1455154 PMC5917440 29707073

[24] Y. Bai , Y. Yang , D. Wang , M. Zhang , Mater. Sci. Eng., A 2017, 703, 116.

[25] S. Shamsdini , S. Shakerin , A. Hadadzadeh , B. S. Amirkhiz , M. Mohammadi , Mater. Sci. Eng., A 2020, 776, 139041.

[26] S. Yin , C. Chen , X. Yan , X. Feng , R. Jenkins , P. O'Reilly , M. Liu , H. Li , R. Lupoi , Addit. Manuf. 2018, 22, 592.

[27] R. Casati , J. Lemke , A. Tuissi , M. Vedani , Metals 2016, 6, 218.

[28] B. Mooney , K. I. Kourousis , R. Raghavendra , Addit. Manuf. 2019, 25, 19.

[29] V. K. Vasudevan , S. J. Kim , C. M. Wayman , Metall. Trans. A 1990, 21, 2655.

[30] W. Xu , P. Rivera‐Díaz‐del‐Castillo , W. Wang , K. Yang , V. Bliznuk , L. Kestens , S. Van der Zwaag , Acta Mater. 2010, 58, 3582.

[31] D. Scotto D'Antuono , J. Gaies , W. Golumbfskie , M. L. Taheri , Acta Mater. 2017, 123, 264.

[32] E. Dontsova , J. Rottler , C. W. Sinclair , Phys. Rev. B 2015, 91, 224103.

[33] A. Mahata , M. A. Zaeem , M. I. Baskes , Modell. Simul. Mater. Sci. Eng. 2018, 26, 025007.

[34] X. Zhao , S. Dong , S. Yan , X. Liu , Y. Liu , D. Xia , Y. Lv , P. He , B. Xu , H. Han , Mater. Sci. Eng., A 2020, 771, 138557.

[35] R. Seede , D. Shoukr , B. Zhang , A. Whitt , S. Gibbons , P. Flater , A. Elwany , R. Arroyave , I. Karaman , Acta Mater. 2020, 186, 199.

[36] J. J. S. Dilip , G. D. J. Ram , T. L. Starr , B. Stucker , Addit. Manuf. 2017, 13, 49.

[37] T. LeBrun , T. Nakamoto , K. Horikawa , H. Kobayashi , Mater. Des. 2015, 81, 44.

[38] T.‐H. Hsu , Y.‐J. Chang , C.‐Y. Huang , H.‐W. Yen , C.‐P. Chen , K.‐K. Jen , A.‐C. Yeh , J. Alloys Compd. 2019, 803, 30.

[39] H. K. Rafi , T. L. Starr , B. E. Stucker , Int. J. Adv. Manuf. Technol. 2013, 69, 1299.

[40] W. Chen , L. Xu , Y. Zhang , Y. Han , L. Zhao , H. Jing , Virtual Phys. Prototyping 2021,17, 366.

[41] H. Asgari , M. Mohammadi , Mater. Sci. Eng., A 2018, 709, 82.

[42] X. Yan , C. Chen , C. Chang , D. Dong , R. Zhao , R. Jenkins , J. Wang , Z. Ren , M. Liu , H. Liao , R. Lupoi , S. Yin , Mater. Sci. Eng., A 2020, 781, 139227.

[43] K. Saeidi , D. L. Zapata , F. Lofaj , L. Kvetkova , J. Olsen , Z. Shen , F. Akhtar , Addit. Manuf. 2019, 29, 100803.

[44] C. Tan , Y. Chew , F. Weng , S. Sui , Z. Du , F. L. Ng , G. Bi , Virtual Phys. Prototyping 2021, S1.

[45] X. Li , Y. H. Tan , H. J. Willy , P. Wang , W. Lu , M. Cagirici , C. Y. A. Ong , T. S. Herng , J. Wei , J. Ding , Mater. Des. 2019, 178, 107881.

[46] E. Jelis , M. Clemente , S. Kerwien , N. M. Ravindra , M. R. Hespos , JOM 2015, 67, 582.

[47] R. Mertens , B. Vrancken , N. Holmstock , Y. Kinds , J. P. Kruth , J. Van Humbeeck , Phys. Procedia 2016, 83, 882.

[48] M. G. Jiang , Z. W. Chen , J. D. Tong , C. Y. Liu , G. Xu , H. B. Liao , P. Wang , X. Y. Wang , M. Xu , C. S. Lao , Mater. Res. Lett. 2019, 7, 426.

[49] Y. H. Wang , J. M. Kang , Y. Peng , T. S. Wang , N. Hansen , X. Huang , Scr. Mater. 2018, 155, 41.

[50] A. Hadadzadeh , A. Shahriari , B. S. Amirkhiz , J. Li , M. Mohammadi , Mater. Sci. Eng., A 2020, 787, 139470.

[51] J. H. Martin , B. D. Yahata , J. M. Hundley , J. A. Mayer , T. A. Schaedler , T. M. Pollock , Nature 2017, 549, 365. 28933439 10.1038/nature23894

[52] W. Bleck , X. Guo , Y. Ma , Steel Res. Int. 2017, 88, 1700218.

[53] S. S. Xu , J. P. Li , Y. Cui , Y. Zhang , L. X. Sun , J. Li , J. H. Luan , Z. B. Jiao , X. L. Wang , C. T. Liu , Z. W. Zhang , Int. J. Plast. 2020, 128, 102677.

[54] B. He , B. Hu , H. Yen , G. Cheng , Z. Wang , H. Luo , M. Huang , Science 2017, 357, 1029. 28839008 10.1126/science.aan0177

[55] C. Tan , J. Zou , D. Wang , W. Ma , K. Zhou , Composites, Part B 2022, 236, 109820.

[56] D. M. Norfleet , D. M. Dimiduk , S. J. Polasik , M. D. Uchic , M. J. Mills , Acta Mater. 2008, 56, 2988.

