# Supplementary material for: Machine Learning Customized Novel Material for Energy‐Efficient 4D Printing
Source: Adv Sci (Weinh). 2023 Feb 5;10(10):2206607. doi: 10.1002/advs.202206607 (PMC10074080; doi:10.1002/advs.202206607)
Supplement: Supplementary file 1 — Supporting Information [file ADVS-10-2206607-s002.pdf]

ADVANCED SCIENCE
Open Access

Supporting Information

for *Adv. Sci.*, DOI 10.1002/advs.202206607

Machine Learning Customized Novel Material for Energy-Efficient 4D Printing

Chaolin Tan, Qian Li, Xiling Yao*, Lequn Chen, Jinlong Su, Fern Lan Ng, Yuchan Liu, Tao Yang*, Youxiang Chew*, Chain Tsuan Liu and Tarasankar DebRoy*

Supporting Information

Machine Learning Customized Novel Material for Energy-efficient 4D Printing

Chaolin Tan [a, *], Qian Li [b], Xiling Yao [a, *], Lequn Chen [a], Jinlong Su [a], Fern Lan Ng [a], Yuchan Liu [a], Tao Yang [b, *], Youxiang Chew [a, *], Chain Tsuan Liu [b], Tarasankar DebRoy [c]

[a] Singapore Institute of Manufacturing Technology, A*STAR, 5 Cleantech Loop, Singapore

[b] Department of Materials Science & Engineering, City University of Hong Kong, Hong Kong, China

[c] Department of Materials Science & Engineering, Pennsylvania State University, University Park, PA, United States

* Corresponding authors:

E-mail addresses: tclscut@163.com (C. Tan), yaox@outlook.com (X. Yao) chewyx@simtech.a-star.edu.sg (Y. Chew), and taoyang6-c@my.cityu.edu.hk (T. Yang).

Supplementary Figures

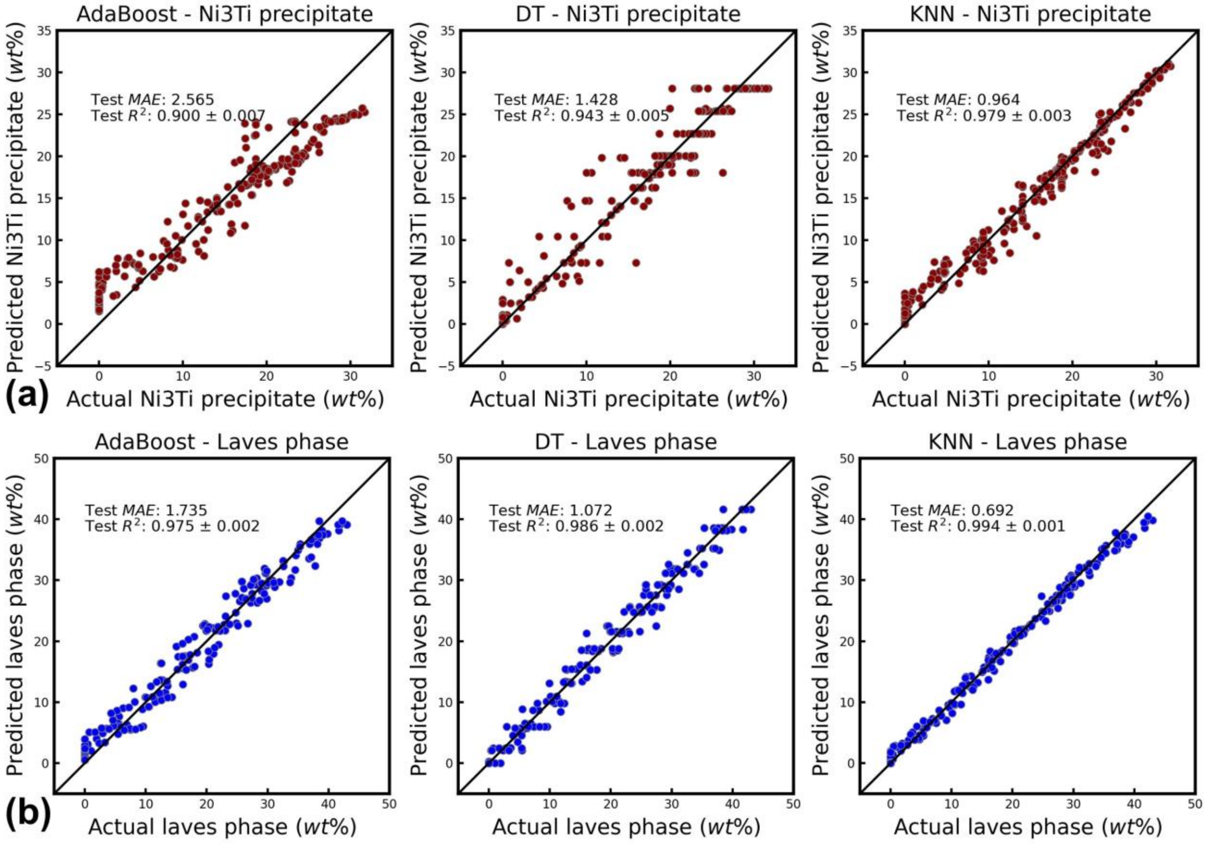

Figure S1. The accuracy of the surrogate models for (a) Ni_3Ti precipitate and (b) Laves phase predicted by Adaptive Boosting (AdaBoost), Decision Tree (DT), K-nearest neighbour (KNN), and Random Forrest (RF) algorithms. The RF algorithm shows the highest R^2 score and lowest MAE value. (Note: the higher R^2 score indicates better performance, while a lower MAE value means better performance.).

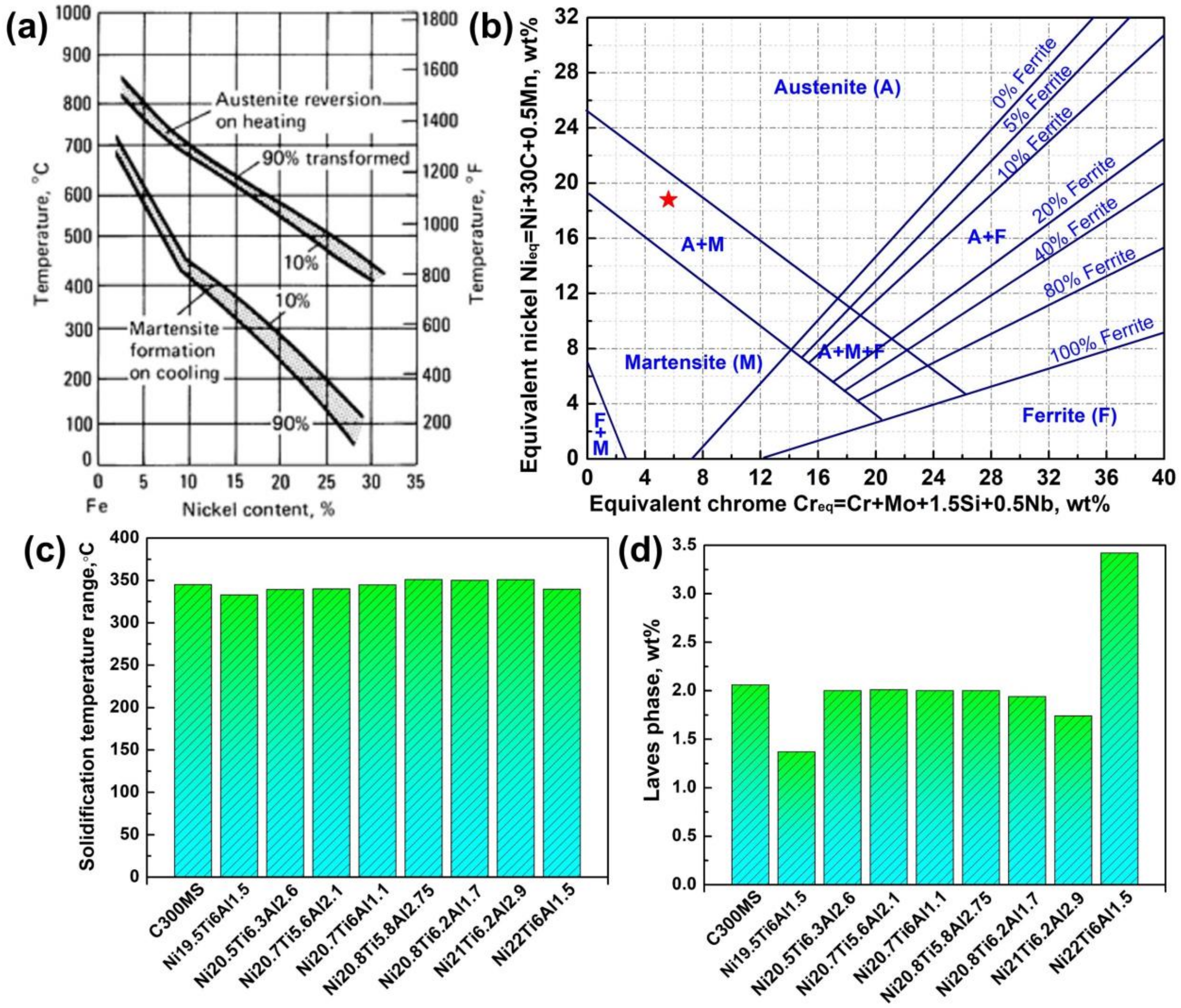

Figure S2. (a) The metastable phase diagram of the Fe-Ni system [1]. (b) Schaeffler-Delong diagram predicts phase compositions via equivalent Ni and Cr (the star "☆" stands for the commercial C300 maraging steel with 18.2 wt% Ni) [2]. (c) Solidification temperature range in materials with different compositions (balance Fe), which shows the Fe-20.8Ni-6.2Ti-1.7Al (wt %) has a similar value with the commercial C300MS. (d) Laves phase contents in materials with different compositions (balance Fe). The designed NMS shows less Laves phase fraction than commercial C300 MS, the higher Ni content (especially when >21 wt%) will increase the content of Laves phase significantly.

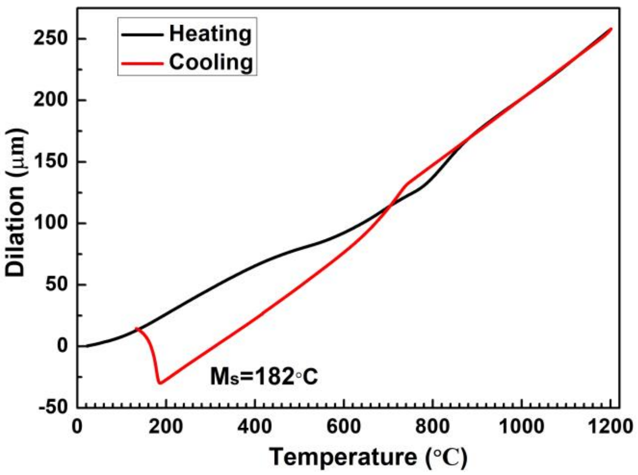

Figure S3. The dilatometer curve of the LDED-processed NMS by ILP deposition strategies, the *Ms* is determined as 182 ºC.

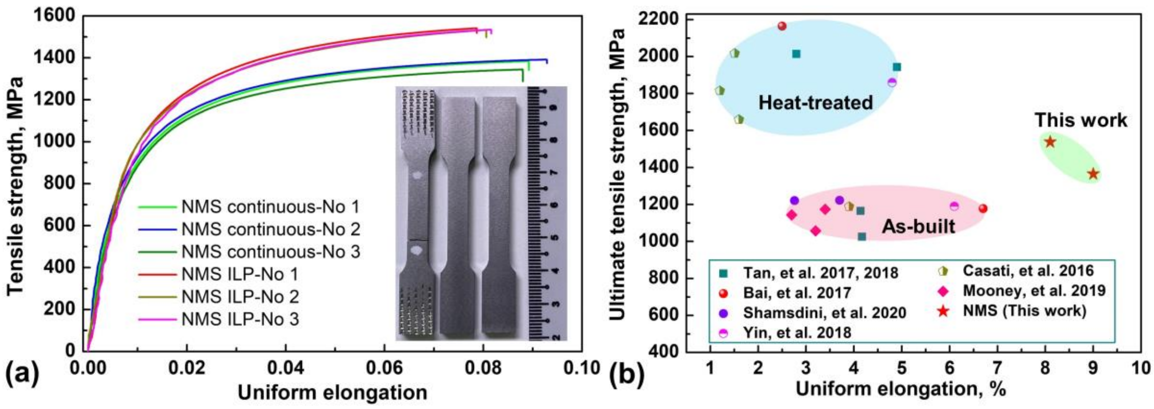

Figure S4. (a) The tensile engineering stress-strain curves of the LDED-processed NMS with continuous and interlayer pause (ILP) deposition strategies (inset photo shows tensile samples with a thickness of 3 mm). (b) Tensile yield strength versus uniform elongation of NMS compared with C300 CMS processed by LAM [1-7].

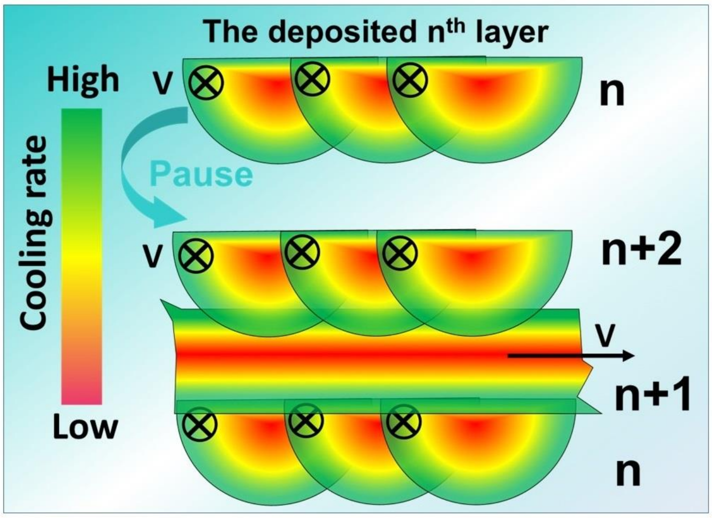

Figure S5. Illustration of cooling rates at different regions with ILP deposition strategy.

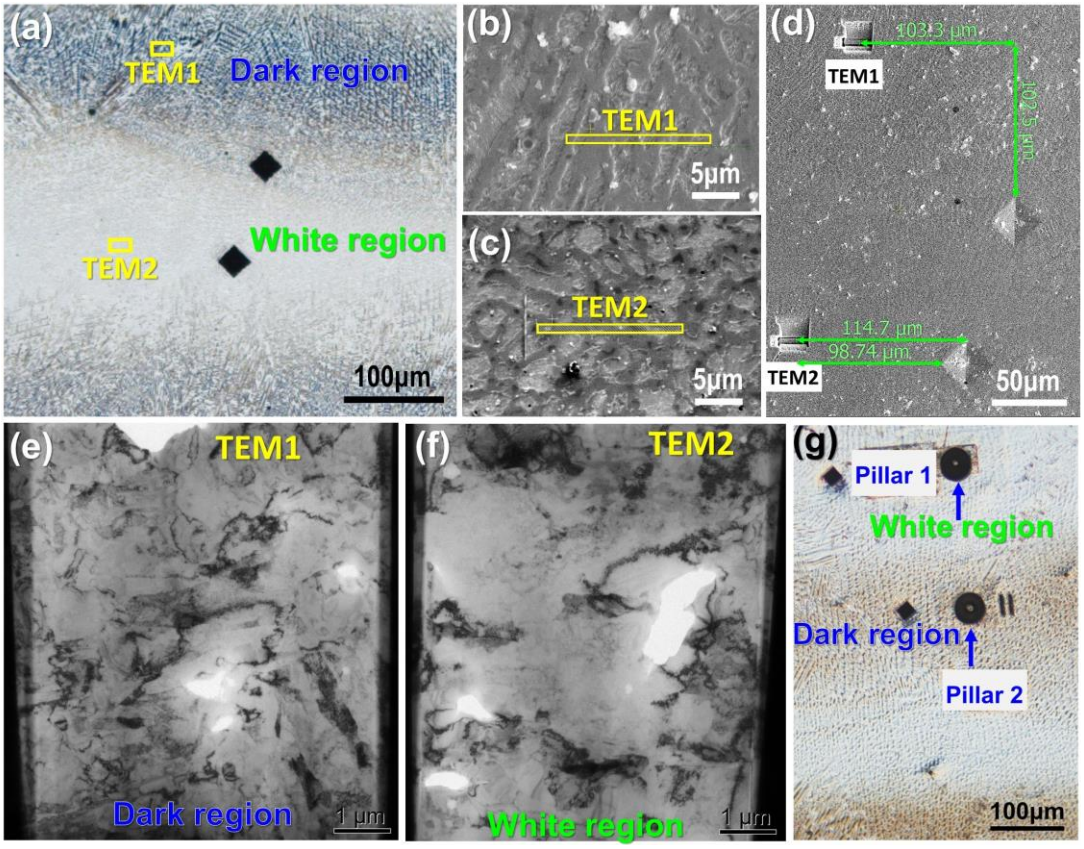

Figure S6. TEM thin foils and micropillars extracted from the dark and white regions of the ILD sample positioned by Vickers indentations. (a) OM image shows the dark and white regions with indentations for positioning, (b) and (c) the microstructures of the FIB extraction regions where the long dendrites are more prevalent in the dark region, (d) FIB extraction regions correlative to the indentations, (e) and (f) TEM thin foils extracted by FIB from the dark and white regions (the dark region shows more dislocations), and (g) OM image shows the micropillars extracted from the dark and white regions with indentations for positioning.

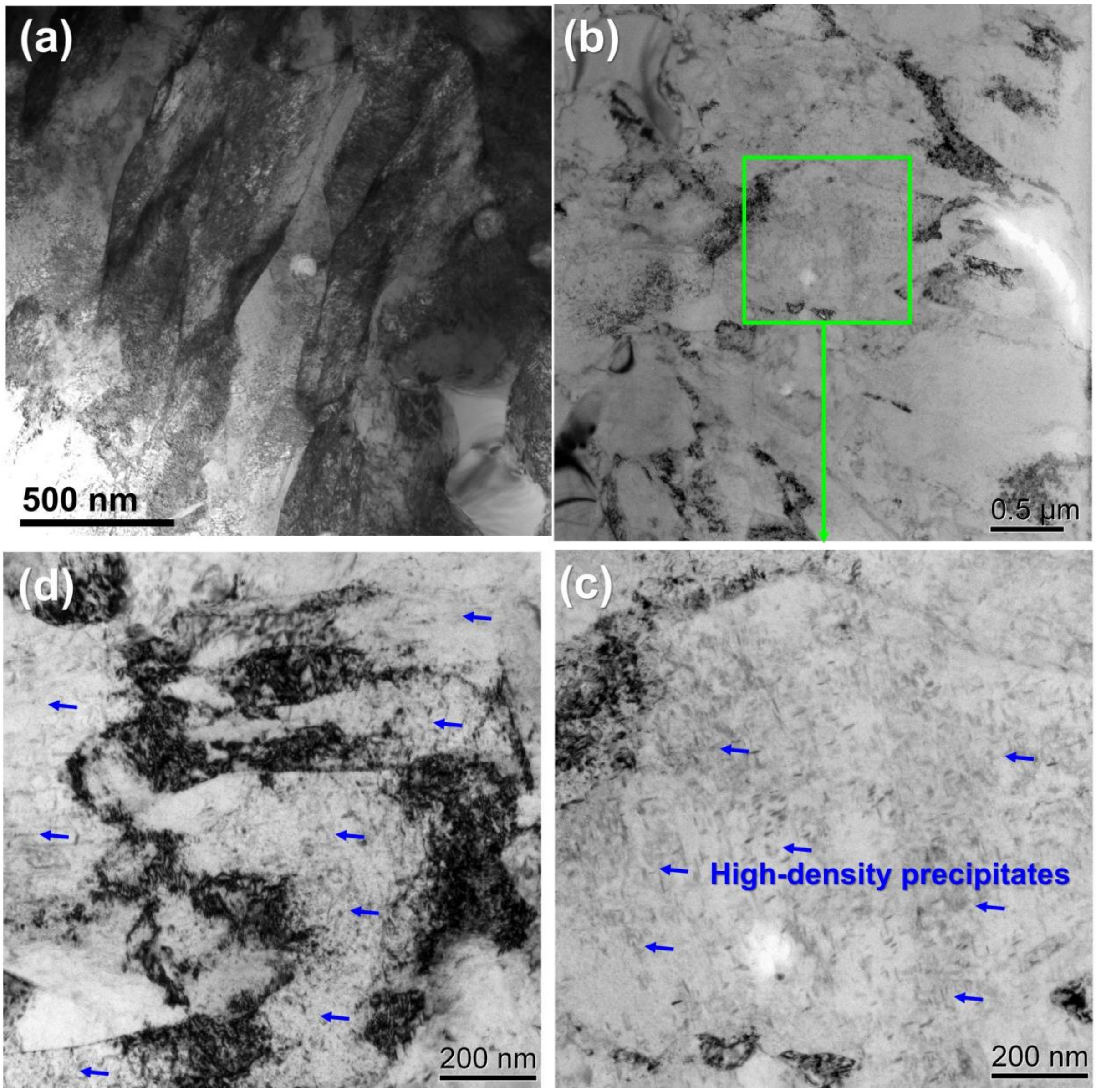

Figure S7. TEM analysis on the bcc-rich dark region of the ILP sample. (a) and (b) low-magnification bright-field (BF) TEM observations, (b) zoom-in BF-TEM image showing high-density precipitates, and (c) BF-TEM showing the high-density precipitates in the dislocation accumulation region.

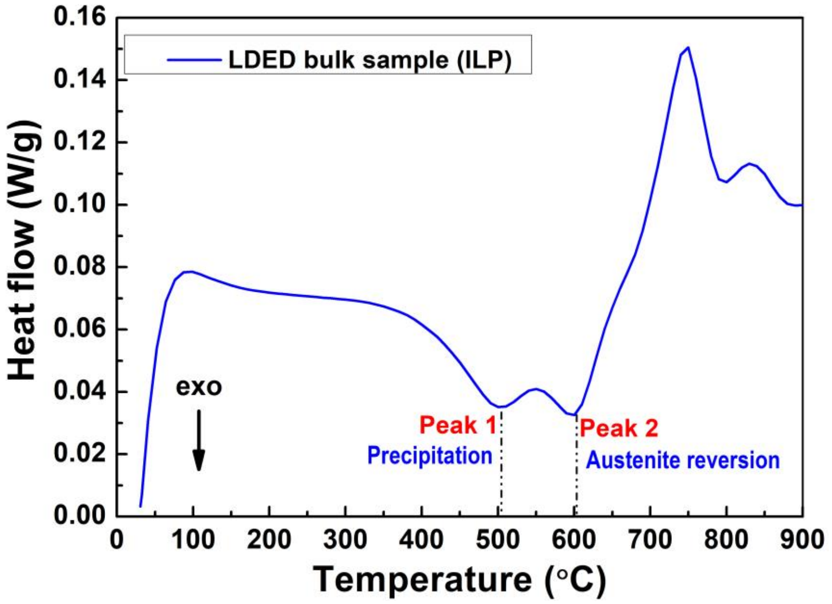

Figure S8. DSC analysis of the LDED processed bulk NMS using ILP deposition strategy. The exothermic peak 1 is representative of precipitations, and the exothermic peak 2 is related to austenite reversion [2, 8].

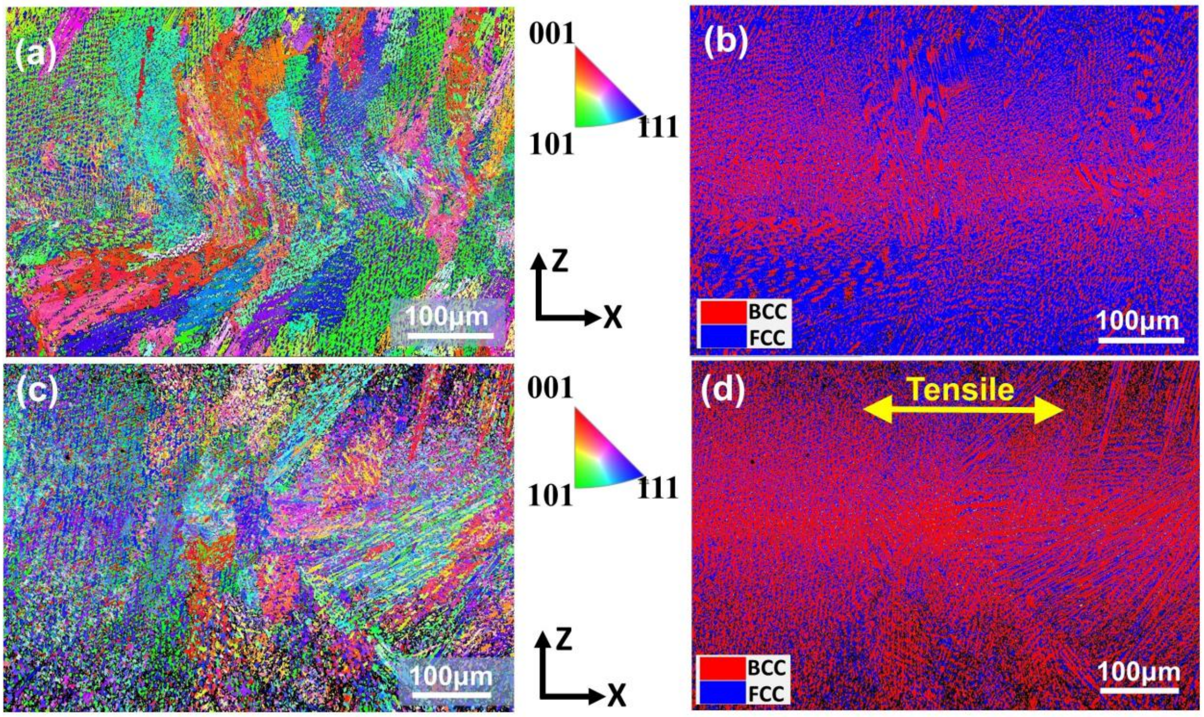

Figure S9. EBSD analysis on the ILP sample before and after tensile test. (a) IPF map and (b) phase distribution map of as-built condition. (c) IPF map and (d) phase distribution map after tensile test, the EBSD scan region is close to the fractured edge.

Supplementary Tables

Table S1. The chemical composition of the NMS (designed value and as-received powder).

**Element** (wt. %)	**Ni**	**Ti**	**Al**	**O**	**C**	**Fe**
Designed	20.8	6.2	1.7	<0.05	0.03	Bal.
As-received powder	21.1	6.3	1.3	0.024	0.012	Bal.

Table S2. A summary of mechanical properties of the LDED-produced NMS.

Sample	Condition	UTS (MPa)	YS (MPa)	Uniform El (%)	Break El (%)	Hardness $HV_{0.1}$
NMS Continuous	As-built	1365 ± 31	824± 15	9.0 ± 0.3	9.0 ± 0.3	408 ± 16
NMS ILP	As-built	1538 ± 5	966 ± 22	8.1 ± 0.2	8.1 ± 0.2	501 ± 17
C300 MS	As-built	1119 ± 23	895 ± 31	5.6 ± 0.3	17.3 ± 0.7	409 ± 26
C300 MS	Heat-treated	1756 ± 7	1612 ± 7	2.3 ± 0.1	5.7 ± 1.5	547 ± 13

Supplementary text

Discussion on the machine learning algorithms for surrogate models

The dataset used for surrogate modelling was obtained from CALPHAD results, which are described in Supplementary Table S3. A total of 1815 data points are used to train the surrogate models, which are regression tasks predicting the Laves phase and Ni_3Ti precipitate content using alloy compositions (Fe, Ni, Ti, Al) as input. We used scikit-learn Python packages [9] to implement four ML algorithms in the surrogate modelling: Decision Tree (DT) [10], K-nearest Neighbour (KNN) [11], Adaptive Boosting (AdaBoost) [12] and Random Forest (RF) [13]. 70 % of the CALPAHD data in DoCE were used for surrogate model training, and the other 30 % were used for testing. The best hyper-parameters for each ML algorithm are found using grid search method [14], which are summarised in Supplementary Table S4. After the hyper-parameter tuning, we used 5-fold cross-validation for validating and comparing the ML model performance. The model accuracy was evaluated based on coefficient of determination (R^2) score, which is defined as:

$$R^2(y,\hat{y}) = 1 - \frac{\sum_{i=1}^{n}(y_i - \hat{y_i})}{\sum_{i=1}^{n}(y_i - \bar{y_i})}$$

where n is the number of the dataset, y_i is the value of i-th data in the dataset, $\hat{y_i}$ is the predicted value of i-th data, and $\bar{y_i}$ is the mean of the data points in the dataset. The performance matric for each ML algorithm is also evaluated by mean absolute error (MAE), which is defined by the following equation:

$$MAE(y,\hat{y}) = \frac{1}{n}\sum_{i=1}^{n}|y_i - \hat{y_i}|$$

The performance for each candidate surrogate model for Laves phase and Ni_3Ti precipitate predictions are shown in Figure 2. The error bars show the standard deviation for each metric based on the trained models, which come from 5-fold cross-validation. The RF algorithm achieves the highest R^2 score (99.5%) and lowest MAE error compared to the other models for both Laves phase and Ni_3Ti precipitate predictions, which exhibits a great predictive capability given a small amount of available data. A comparison between the predicted and actual values of the Laves phase and Ni_3Ti precipitates in the test set partition is shown in Supplementary Figure S2. When a model has an R^2 of 1, all the points would be on the diagonal line, which means a perfect fit. Thus, our regression results for the RF are reliable and capable of predicting accurate values close to the actual values of Laves phase and Ni_3Ti precipitate contents given the alloy composition.

Table S3. Dataset for surrogate modelling.

Phase content	Dataset	Input	Tasks	Rec - metrics	Algorithm
Laves phase	1815	Alloy Composition (Ti, Ni, Al)	regression	R^2 - MAE	RF
Ni_3Ti content	1815	Alloy Composition (Ti, Ni, Al)	regression	R^2 - MAE	RF

Table S4. Hyperparameters and their range studied in ML for Ni_3Ti precipitate and Laves phase surrogate modelling.

Classifiers	Hyperparameters	Optimal values	Range studied
KNN	Number of neighbours	6 (Ni_3Ti precipitate)	3-10
		8 (Laves phase)	
AdaBoost	Number of estimators	400 (Ni_3Ti precipitate)	[100,200,300,400]
		300 (Laves phase)	
DT	Maximum depth	6 (Ni_3Ti precipitate)	[2,3,4,5,6]
		6 (Laves phase)	
RF	Maximum depth of tree	100 (Ni_3Ti precipitate)	[10,30,60,90,100]
		60 (Laves phase)	

Discussion on the alloy composition optimization

A population-based metaheuristic algorithm, i.e., Differential Evolution (DE) [15], was used to solve the composition optimization problem described by the formulation shown in Supplementary Figure S10. Differential evolution is a stochastic population-based optimization method that iteratively improves a candidate solution with respect to a given measure of quality. This is a derivative-free optimization approach that makes no assumptions about the problem being optimized, allowing it to search very large spaces of candidate solutions for global optimization problems. We used SciPy Python library to implement the DE algorithm[16]. The specifications of the algorithm parameters are listed in Supplementary Table S5.

Table S5. Parameters used in Differential Evolution algorithm.

**Parameter**	**Value**
Strategy	'best1bin'
maxiter	100
popsize	15
tol (relative tolerance)	0.01
atol (absolute tolerance)	0
Mutation (differential weight, F)	(0.5, 1)
Recombination (cross-over probability)	(0.7)
'init' (type of initialisation)	'latinhypercube'
Updating method	'immediate'

The algorithm was solved iteratively with 100 iterations. At each pass through the population, four steps were executed:

- **[Step 1]**: Initialization of population: Latin Hypercube sampling is used to generate the population, to cover as much of the available parameter space as possible.
- **[Step 2]**: Mutation: after initializing the parameters, the DE algorithm mutates each candidate solution by mixing it with other candidate solutions to produce a trial candidate. The 'best1bin' strategy was selected to compute the mutation. In this strategy, two members of the population are randomly chosen (i.e., $X[rand0]$ and $X[rand1]$). Their difference is used to mutate the best member (X_{best}) in the current iteration:

$$X' = X_{best} + F(X[rand0] - X[rand1])$$

where F is the mutation constant (also known as differential weight), and X' denotes the resultant trial vector.

- **[Step 3]**: Cross-over (recombination): The trial vector X' and the original vector X_{best} are then mixed (recombined), based on the cross-over probability. Beginning with a randomly chosen i-th parameter, the new trial vector is sequentially filled (in modulo) with parameters from X' or the original candidate X_{best}. A binomial distribution is used to determine whether to use X' or the X_{best}.
- **[Step 4]**: Replacement and Evaluation: after generating the new trail vector X', it is evaluated by the fitness function. If the trial is better than the original candidate then it takes its place. If it is also better than the best overall candidate it also replaces that.

```
Alloy composition optimization problems
Input: Alloy composition:
        Ni(wt%):x1;
        Ti(wt%):x2;
        Al(wt%):x3;
Objective: Maximising Ni3Ti precipitate: f1(x1,x2,x3);
           Minimising Laves phase content: f2(x1,x2,x3)
Constraint:
            (1) x3 < x2;
            (2) f2(x1,x2,x3)<5,wt%;
            (3) 18 < x1 < 21;
            (4) 5 < x2 < 10;
            (5) 1 < x3 < 5;
•  f1(x1,x2,x3) is the surrogate model for Ni3Ti precipitate by RF algorithm.
•  f2(x1,x2,x3) is the surrogate model for Laves phase by RF algorithm.
```

Figure S10. Alloy composition optimization problem.

References

[1] C. Tan, K. Zhou, W. Ma, P. Zhang, M. Liu, T. Kuang, Materials & Design 134 (2017) 23-34.

[2] C. Tan, K. Zhou, M. Kuang, W. Ma, T. Kuang, Science and Technology of Advanced Materials 19 (2018) 746-758.

[3] Y. Bai, Y. Yang, D. Wang, M. Zhang, Materials Science and Engineering: A 703 (2017) 116-123.

[4] S. Shamsdini, S. Shakerin, A. Hadadzadeh, B.S. Amirkhiz, M. Mohammadi, Materials Science and Engineering: A 776 (2020) 139041.

[5] S. Yin, C. Chen, X. Yan, X. Feng, R. Jenkins, P. O'Reilly, M. Liu, H. Li, R. Lupoi, Additive Manufacturing 22 (2018) 592-600.

[6] R. Casati, J. Lemke, A. Tuissi, M. Vedani, Metals 6 (2016) 218.

[7] B. Mooney, K.I. Kourousis, R. Raghavendra, Additive Manufacturing 25 (2019) 19-31.

[8] S. Amirabdollahian, F. Deirmina, L. Harris, R. Siriki, M. Pellizzari, P. Bosetti, A. Molinari, Scripta Materialia 201 (2021).

[9] F. Pedregosa, G. Varoquaux, A. Gramfort, V. Michel, B. Thirion, O. Grisel, M. Blondel, P. Prettenhofer, R. Weiss, V. Dubourg, the Journal of machine Learning research 12 (2011) 2825-2830.

[10] S.R. Safavian, D. Landgrebe, IEEE transactions on systems, man, and cybernetics 21 (1991) 660-674.

[11] S.A. Dudani, IEEE Transactions on Systems, Man, and Cybernetics (1976) 325-327.

[12] Y. Freund, R.E. Schapire, Journal of computer and system sciences 55 (1997) 119-139.

[13] L. Breiman, Machine learning 45 (2001) 5-32.

[14] S.M. LaValle, M.S. Branicky, S.R. Lindemann, The International Journal of Robotics Research 23 (2004) 673-692.

[15] R. Storn, K. Price, Journal of global optimization 11 (1997) 341-359.

[16] P. Virtanen, R. Gommers, T.E. Oliphant, M. Haberland, T. Reddy, D. Cournapeau, E. Burovski, P. Peterson, W. Weckesser, J. Bright, Nature methods 17 (2020) 261-272.
